# Links Between Variation in Movement-Based Visual Signals and Social Communication Complexity in an Asian Agamid Lizard *Phrynocephalus vlangalii*

**DOI:** 10.3390/ani15010038

**Published:** 2024-12-27

**Authors:** Jian Liu, Qiaohan Hu, Yin Qi

**Affiliations:** 1 Chengdu Institute of Biology, Chinese Academy of Sciences, Chengdu 610041, China; liujian@cib.ac.cn (J.L.); huqiaohan@mail.kiz.ac.cn (Q.H.); 2 University of Chinese Academy of Sciences, Beijing 100049, China; 3 State Key Laboratory of Genetic Resources and Evolution, Kunming Institute of Zoology, Chinese Academy of Sciences, Kunming 650223, China; 4 Key Laboratory of Genetic Evolution & Animal Models, Kunming Institute of Zoology, Chinese Academy of Sciences, Kunming 650223, China

**Keywords:** animal communication, social complexity, movement-based visual signals, parental care, *Phrynocephalus vlangalii*

## Abstract

The social complexity hypothesis suggests that complex social interactions drive the evolution of sophisticated communicative signals. While the relationship between social communication and the complexity of sound and color signals has been extensively studied, the correlation between social communication and movement-based visual signal complexity remains underexplored. In this study, we selected the Asian agamid lizard, *Phrynocephalus vlangalii*, as our model system. Through a combination of controlled experiments, behavioral observations, and signal quantification, we examined the relationship between social communications and variation in movement-based visual signals and tested our social complexity hypothesis. Our experiments revealed that males significantly decreased the tail coil duration during the mating season to deal with high social interaction. Conversely, females significantly increased the tail display duration and variation in mean tail coil amplitude in line with the intensity of parental care, and tail display duration showed a significant and positive correlation with female reproductive investment. These findings suggest that social communication plays an important role in shaping the changes in movement-based visual signals, providing new evidence for the social complexity hypothesis.

## Simple Summary

Clarifying the relationship between signal variation and social interactions is important for understanding the evolution of signal complexity. While sound and color signals have been well studied, less is known about how social communication shapes the movement-based visual signals. In this study, we examined the relationship between the variation in tail displays and social interactions in the Asian agamid lizard *Phrynocephalus vlangalii*. We found that males significantly reduced the duration of tail coiling during the mating season, while females increased both the duration and variation in tail movements to meet the demands of parental care. Additionally, females that invested more in reproduction displayed their tails for longer periods. These findings demonstrate that social interaction influences movement-based visual signals, providing new evidence for the evolution of signal complexity from a new signal pattern.

## 1. Introduction

Animal social communication depends on a variety of signals. The signaler influences behavioral response of the recipient through signal change, and its behavioral response may impact the signal structure accordingly, thereby forming a complete communication loop [1]. Nevertheless, the signal structure is not fixed. Signalers likely adjust their signals according to specific situations, such as climate change [2], season variation [3], and environment noise [4,5,6], so as to ensure the signal efficacy under background noise and physiological constraint [7]. For example, in the context of climate change, the male spadefoots (*Spea multiplicata)* reduced their call speed in response to the colder environment [8]. The neotropical lizard *Liolaemus pacha* (Iguania: Liolaemidae) exhibits more frequent tongue flicking and visual displays during the breeding season [9]. The tropical *Anolis* lizards have evolved diverse visual signaling strategies to resist interference from environment noise, such as visual motion and wind-blown vegetation [10].

Recently, the social complexity hypothesis focusing on the relationship between signal complexity and social complexity has garnered a significant attention due to its intimate connection with human language [11,12,13,14]. The hypothesis posits that the complexity of social communication drives the evolution of signal complexity, and that maintaining complex social structures necessitates complex signals [15,16]. This hypothesis has been evidenced across numerous animal groups [17,18,19,20,21]. For example, in macaque taxa, species experiencing complex social interactions exhibit greater vocal diversity and flexibility [22]. In birds, species that depend on cooperative reproduction display a larger sound repertoire [23]. Living in groups promotes the differentiation of sound signals in striped dolphins between different ages and sexes [24]. Movement-based visual signals (e.g., claw wave in fiddler crab, head bod in *Anolis* lizard) have been widely used in many species [20,25,26] and play important roles in social communication. Nevertheless, whether the relationship between the variation in movement-based display signals and complexity of social interactions is in line with social complexity hypothesis remains largely unclear.

The Asian agamid lizard, *Phrynocephalus vlangalii*, serves as an excellent model system to study the relationship between the variation in movement-based visual signals and social communication complexity. This species primarily communicates through movement-based visual signals (tail coiling, tail lashing) during male–male competition, male courtship, and female territorial defense [27]. More importantly, a lot of research has shown that *P. vlangalii* can adjust the tail coiling and tail lashing according to social context. For example, the female resident would display toward the intruder female more quickly when a neighboring male is present, indicating the mate defense role of female movement-based visual signals [28]. The resident male would adjust their response according to the tail coil and tail lash speed of intruder [29]. In addition, the movement-based visual signals in *P. vlangalii* encode information on individual physical condition and burrow quality [30]. More interestingly, when the offspring are born, they live in the female burrow for a while and female aggression increases accordingly [31], suggesting the burrow share functions in parental care, and the duration of burrow sharing provides a nice indicator of parental care investment. These studies provide important perspectives on the links between movement variation and social communication.

Here, we used *P. vlangalii* as a model system to examine the links between the variation in movement-based visual signals and the complexity of social communication. If the complexity of social interactions indeed influences the variation in movement-based visual signals, we predict that the variation in these signals is higher during the mating season compared to the non-mating season for males, due to the frequent male–male competition and male courtship. For females, we predict that the variation in movement-based visual signals is associated with investment in reproduction, such as energy and parental care: individuals with high reproductive investment are likely to exhibit more variable signals.

## 2. Materials and Methods

### 2.1. Study Site and Species

Our study was conducted near the Xiaman field station of Zoige Wetland Nature Reserve, in southwestern China (33°43′25.0″ N, 102°29′04.0″ E, with an elevation of 3475 m a.s.l.). The climate in this area is characterized by drought, low temperature, and strong sunshine, with average monthly precipitation of 54.88 ± 47.99 mm, average monthly minimum temperature of −5.2 ± 8.7 °C, and average monthly sunshine duration of 12.2 ± 1.6 h (data from Weather Atlas). The population density of *P. vlangalii* in this area is approximately 0.1128 ± 0.0172 lizards per square meter [32], mainly distributed around sand dunes. The active season of the lizards lasts from late April to early September, with the mating season in May and June, and young emerging in late August and early September [33].

### 2.2. Male Display Collection

**Figure 1:**
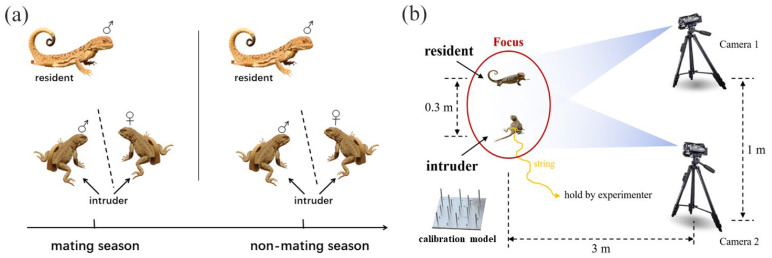
Male experimental scheme design. (**a**) Collection of movement-based visual signals in males. ♂ represents male individuals, while ♀ represents female individuals. (**b**) Schematic of collecting method on movement-based visual signals.

To test the relationship between the variation in movement-based visual signals and the complexity of male social interaction, we employed the most recent 3D method and collected the movement-based visual signals of males both in mating and non-mating seasons from 2016 to 2018 [30]. To examine whether the relationship is variable with social contexts, each resident male experienced two social contexts, including male–male competition and male courtship. For the male–male competition context, we presented focal resident males with an intruder male, while for male courtship context, we presented focal resident males with an intruder female. We waited at least 30 min before presenting the next intruder, based on the natural display rates of one every 20 min (Figure 1a). To facilitate size matching, we first captured the resident males with a lasso, measured their snout–vent length (SVL) and tail length (TL) separately to the nearest 0.01 mm using a vernier caliper (PD-151, Mitutoyo Corporation, Kawasaki, Japan), and weighed their mass to the nearest 0.01 g using an electronic balance (ES-08B, Want Balance Instrument Co., Ltd., Guangzhou, China). To facilitate individual identification, we marked them using a nontoxic paint pen and returned them to the original burrow with a mark flag planted near the burrow entrance. To avoid the potential impact of previous social interactions, individuals used as intruders were collected at least three kilometers away from the habitat of residents, and their burrows were also marked to facilitate subsequent releasing. The snout–vent length (SVL) and tail length (TL) of the intruders were measured using the similar method.

After at least 24 h, we went back to the marked residents and collected the display signals. As shown in Figure 1b, we first confirmed the presence of the resident using binoculars; when the resident was locked down, we immediately set up two cameras (50 frames per second, Sony HDR PJ670, Sony Corporation, Tokyo, Japan) at 3 m away from the resident. The distance between the two cameras was kept at least 1 m apart. We waited for at least 10 min for the resident to acclimatize before commencing filming. At the same time, the other experimenter introduced the intruder from 3 m away using a fishing rod. The intruder was tethered to the rod using 30 cm of dental floss tied around the waist. We terminated a trial after a resident completed its display or before the interaction between resident and intruder escalating into fight. We also terminated a trial if a resident did not exhibit a display after 10 min of presenting an intruder. Before we turned off the camera, a calibration object was placed in the site where the signal from the tail of the resident was displayed for subsequent spatial digitization. We minimized the size difference between the intruder and resident by size-matching. To avoid the effects of physiological stress, each intruder was used a maximum of three times and then released back into their original burrow.

### 2.3. Female Display Collection and Reproductive Investment Measurement

To test the relationship between the variation in movement-based visual signals and female reproductive investment, we collected movement-based visual signals from postpartum females in August 2021. To confirm pregnancy, we constructed a plot (50 m × 50 m) in the field from late July and the pregnancy status of the females was checked daily. When the female showed obvious signs of pregnancy, we captured 30 pregnant females using a lasso, measured their snout–vent length (SVL, to the nearest of 0.01 mm) and mass (to the nearest of 0.01 g), marked them using a nontoxic paint pen, and returned them to the original burrow with a color flag planted nearby the burrow entrance.

We then followed the 30 pregnant females and observed their reproductive status using binoculars. Each lizard was checked twice a day from 9:00 am to 4:00 pm to accurately determine the delivery time. To estimate the energetic investment, we measured the mass of the females after giving birth, calculated the female mass variation before and after postpartum, and used the ratio between female mass variation and mass after postpartum as an estimate of female energetic investment [34,35]. Three days after the female gave birth, we collected the female movement-based visual signals by presenting an intruder female to postpartum females using the same method as the males. We size-matched the resident and intruders and returned the intruders after three trials.

After the display collection on postpartum females, we continued the field observation twice a day to determine the duration that females and babies shared the burrow. When the females and offspring were no longer in the same burrow, we recorded this day as the separation time. Three days of extra observation were conducted to ensure the accuracy of the separation time. We then used the number of days the females and offspring shared the burrow as an estimate of female parental care.

### 2.4. Display Digitization and Variation Estimation

The process of display digitization was consistent with the protocol proposed by Peters et al. [27]. Briefly, we used the video editing software Adobe Premiere Pro CC 2018 (version 12.0) to extract high-quality display clips from the original video and obtained a screenshot containing the calibration objects. The video was then calibrated using Direct Linear Transformation (DLT) in MATLAB 2016b (MathWorks Inc., Natick, MA, USA) with the calibration object screenshot. The 3D coordinates of the two points of the tail base and tail tip were digitized frame by frame for tail display reconstruction. Movements of the tail coil and tail lash were reconstructed for males, while movements of the tail coil were reconstructed for females. Using the reconstructed positional data, we extracted the average tail coil speed, tail coil duration, average tail coil amplitude, average tail lash speed, tail lash duration, and volume of space swept by the tail lash display (VT) for males. The average tail coil (or lash) speed was defined as the average distance moved by the tail tip per second in each display bout; tail coil (or lash) duration was defined as the total time of tail coil (or lash) in each display bout; and average tail coil amplitude was defined as the average distance between the tail tip and tail base. The smaller the average tail coil amplitude, the more tightly coiled the tail. VT was defined as the volume of space swept by the tail tip during lashing in each display bout [30]. Occasionally, there was a time interval between two successive display segments, and we regarded those two display segments as one display segment if the time interval was less than 2 s; otherwise, we classified them as two display segments. When digitizing the female signal, we only extracted the average tail coil speed, tail coil duration, and average tail coil amplitude because tail lash frequency was very low. Very short tail lash was found in five videos of the females; we included them in the overall display duration. Therefore, the female tail display duration used in subsequent analysis was the overall display duration, and the average duration was the ratio of the overall display duration to the number of signal segments. The variation in movement-based displays was estimated using the coefficient of variation (CV), calculated as the standard deviation divided by the mean [36,37], as well as the tail coil (lash) duration.

### 2.5. Statistical Analysis

All statistical analyses were conducted using the R version 4.3.2. The linear mixed models, constructed using the *lmer* function in lme4 package (version 1.1.35.1), were used to analyze the relationship between the variation in male movement-based visual signals and seasons [38]. Variables, including the tail coil duration after logarithmic transformation, CV of tail coil duration, CV of mean tail coil amplitude, CV of maximum tail coil speed, CV of mean tail coil speed, tail lash duration after logarithmic transformation, CV of tail lash duration, CV of tail lash volume, CV of maximum tail lash speed, and CV of mean tail lash speed, were considered dependent variables, while the season and social communication context were included as independents, and a Gaussian error distribution was assumed. In addition, we considered the resident’s SVL, body temperature, and mass as covariates to account for potential effects of size and physiological condition.

The linear model constructed using the *lm* function in the stats package was also used to test the relationship between the variation in movement-based visual signals and reproductive investment in postpartum females [39,40]. Variables, including tail display duration after logarithmic transformation, CV of display duration, CV of mean tail coil amplitude, CV of maximum tail coil speed, and CV of mean tail coil speed were considered as dependent variables, female energetic investment and parental care investment were used as independent variables, and a Gaussian error distribution was assumed. In addition, the resident’s SVL and body temperature were included as covariates to explain the potential impacts of size and physiological condition.

To visualize the relationship between the variation in male movement-based visual signals and seasons, and the relationship between the variation in movement-based visual signals and female parental care investment and energetic investment, we predicted the relationship based on the full model using the *ggplot* function in the *ggplot2* package (version 3.5.1) [41].

## 3. Results

A total of 48 males were collected for movement-based visual signal analysis, including 29 individuals during the mating season and 19 during the non-mating season. Quantification of these signals revealed 380 movement-based events recorded during the mating season, averaging approximately 13 events per individual. In the non-mating season, 210 movement-based events were recorded, with an average of 11 events per individual. See the male movement-based visual signals in Video S1. Parental care behavior was followed and quantified in 30 pregnant females, with 14 giving birth and their parental care being measured. Of the 14 females with recorded parental care data, movement-based visual signals were collected from 12 individuals.

### 3.1. Differences in Variation in Male Movement-Based Visual Signals Between Mating and Non-Mating Seasons

**Figure 2:**
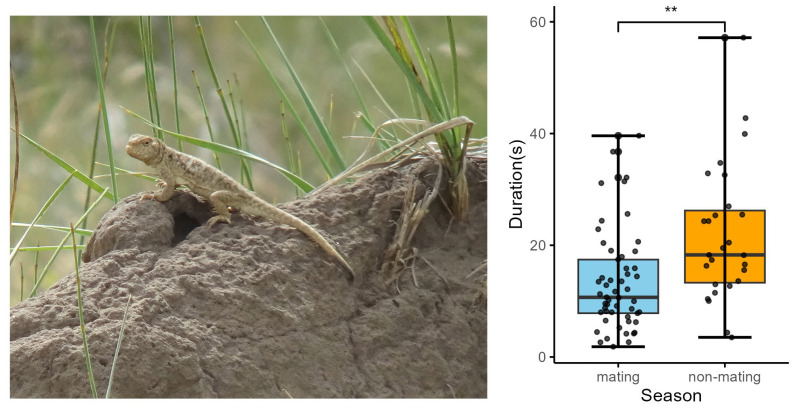
Male image of *P. vlangalii* and the variation in tail coil duration in the male’s movement-based visual signals across seasons. Blue represents the breeding season, and orange represents the non-breeding season. Statistical analysis showed significant differences, with *p* values indicated by *p* < 0.01 (**).

We found a significant change in tail coil duration between the mating and non-mating seasons. The tail coil duration was significantly shorter in the mating season compared with that in the non-mating season (*p* = 0.006; Table S1; Figure 2). No significant change was found in the CV of tail coil (or lash) duration, CV of mean tail coil amplitude, CV of maximum tail coil (or lash) speed, CV of mean tail coil (or lash) speed, CV of VT, or CV of lash duration between the two seasons (Table S1).

### 3.2. Relationship Between Variation in Female Movement-Based Visual Signals and Parental Care

**Figure 3:**
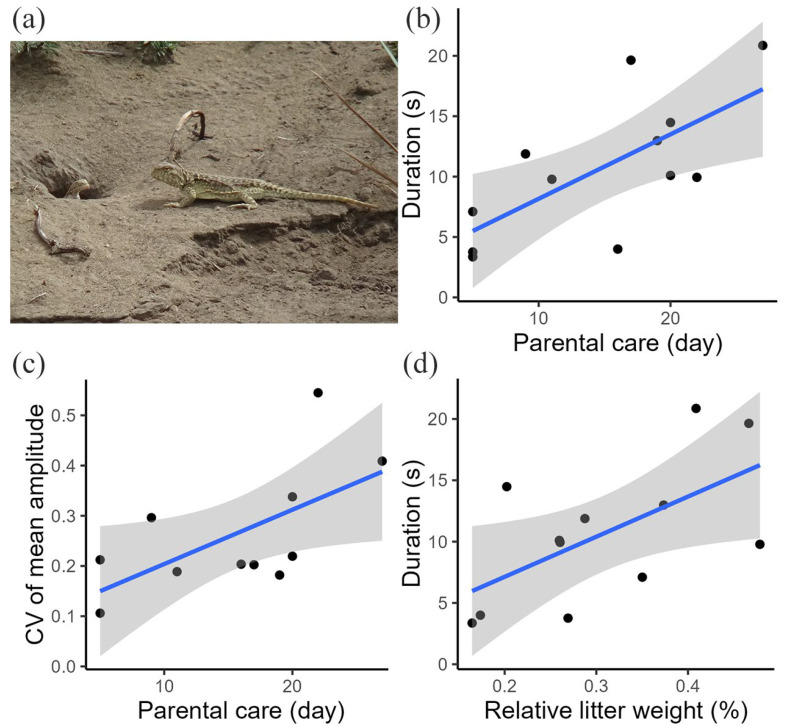
(**a**) Image of a postpartum female and her offspring. (**b**) Relationship between the tail display duration and parental care. (**c**) Relationship between the CV of the tail coil amplitude and parental care. (**d**) Relationship between the tail display duration and reproductive investment. Blue lines represent the fitted regression lines, gray color indicates the confidence intervals, and black dots represent individual data points.

We found a link between the variation in female movement-based visual signals and parental care for postpartum females. The general tail display duration (*p* = 0.025; Table S2; Figure 3b) and CV of mean tail coil amplitude (*p* = 0.047; Table S2; Figure 3c) all increased with parental care time. No significant relationships were found between the CV of the maximum tail coil speed and CV of the mean tail coil speed and parental care (Table S2).

### 3.3. Relationship Between Variation in Female Movement-Based Visual Signals and Energetic Investment

We found a link between the variation in female movement-based visual signals and energetic investment for postpartum females. The general tail display duration increased significantly with energetic investment (*p* = 0.045; Table S3; Figure 3d), suggesting the role of reproductive investment in the variation in tail displays. We found no significant relationships between the CV of tail display duration, CV of mean tail coil amplitude, CV of maximum tail coil speed, CV of mean tail coil speed, and reproductive investment (Table S3).

## 4. Discussion

In this study, we examined the relationship between the variation in movement-based visual signals and social communication and tested the social complexity hypothesis from movement-based signals. Our data revealed that males significantly decreased tail coil duration during the mating compared with the non-mating season. Conversely, females significantly increased tail display duration and the CV of mean tail coil amplitude in line with the intensity of parental care, and tail display duration showed a significant positive correlation with female energetic investment, suggesting it is social communication that drives the variation in movement-based visual signals, providing new evidence for the social complexity hypothesis.

Males decreased tail coil duration during mating season, which is surprising because signal duration is generally considered an important indicator of signal complexity [22,37,42,43]. Typically, higher social complexity correlates with longer signal durations. For example, in some anurans like *Hyla cinerea*, males increase the duration and complexity of their calls in more competitive environments [44]. There is multiple evidence on the links between sexual selection pressure and signal complexity [45,46,47,48]. For example, in a study of male *Physalaemus pustulosus* (túngara frogs), females showed a preference for complex acoustic calls over simple ones [49]. However, we found that the tail coil duration in *P. vlangalii* during the mating season was significantly shorter than in non-mating seasons, which contradicts our expectations considering the influence of social complexity on signal complexity [16,21,50,51,52]. There are two potential explanations. First, males likely reduce tail coil duration to achieve high tail coil frequency to respond to frequent social interaction, such as male–male competition and male courtship [53,54]. This is evidenced by a lot of existing research, for example, higher frequency display instances enhance the chances of successful mate acquisition [55,56,57,58,59]. Alternatively, the shorter tail coil displays may reflect a trade-off between signal complexity and social interactions [60,61]. Males likely choose to decrease the tail coil duration to ensure the energy requirement for male–male competition and male courtship. Multiple intermittent displays allow males to navigate various social scenarios during the mating season [54,60]. There is also multiple evidence on this; for example, studies indicate that the evolution of calling songs in Gomphocerinae (Orthoptera, Acrididae), which involves changes in echeme duration and increased syllable complexity, facilitates the development of signals toward greater complexity [62].

More interestingly, we found obvious links between the variation in movement-based signals and female parental care. Individuals with prolonged parental care and high energetic investment exhibit more complex movement-based signals. This provides a novel insight into the social complexity hypothesis. Parental care is very rare in lizards, and, more importantly, movement-based signals are mainly observed in males of other species. For example, male lizards often exhibit courtship-related behaviors such as head bobbing and push-ups [63,64,65]. The relationship between the variation in movement-based signals (e.g., duration and amplitude) and parental care suggests the applicability of the social complexity hypothesis to females. Postpartum females have to navigate various complex social scenarios, such as territory defense and offspring protection, while also recovering their physiological condition as quickly as possible. In this context, variable visual signals likely facilitate mutual evaluations during social interactions. A similar pattern has been found in *Liolaemus pacha* lizards, where visual displays are crucial for assessing social dynamics [66]. Additionally, we also found a link between the variation in movement-based signals and female energetic investment. This may further strengthen the relationship between female reproductive investment and the variation in movement-based signals, because female energetic investment and parental care are important indicators of female reproductive investment, which in turn can influence offspring survival and fitness [67,68]. This may also promote the iterative inheritance of movement-based signals, as demonstrated in studies of behavioral adaptations and signal evolution in response to environmental and social pressures [69,70].

## 5. Conclusions

In summary, our study provides new insights into the universality of the social complexity hypothesis from movement-based visual signals, although the relationship between the variation in movement-based signals and social complexity is different between the sexes. Males likely achieve the signal complexity by reducing the signal duration, but females tend to adjust the amplitude and increase the signal duration so as to respond to different social scenarios. This fully shows that variation in movement-based display depends on specific social interaction and the life-history stage, thereby enhance our understanding of the factors driving signal complexity. Much more specific research should be carried out to elucidate the efficacy of different signal variations under different social contexts, and adaptation of male shorter displays in mating season should be figured out.

## Data Availability

Data that support the findings of this study have been deposited in figshare at https://doi.org/10.6084/m9.figshare.27641712.v1.

## Supplementary Materials

The following supporting information can be downloaded at https://www.mdpi.com/article/10.3390/ani15010038/s1. Table S1: The results of the general linear model summarized the relationship between the variation in male movement-based display and the season in male *P. vlangalii* lizards; Table S2: The results of the general linear model summarized the relationship between the postpartum female’s variation in movement-based display and parental care time in *P. vlangalii* lizards; Table S3: The results of the general linear model summarized the relationship between the postpartum female’s variation in movement-based display and reproductive investment in *P. vlangalii* lizards; Video S1: A segment of male movement-based visual signals.
