# Supplementary material for: Links Between Variation in Movement-Based Visual Signals and Social Communication Complexity in an Asian Agamid Lizard Phrynocephalus vlangalii
Source: Animals (Basel). 2024 Dec 27;15(1):38. doi: 10.3390/ani15010038 (PMC11718965; doi:10.3390/ani15010038)
Supplement: Supplementary file 1 [file animals-15-00038-s001.zip › Supplementary Materials/Table S1-3.pdf]

Table S1. The results of the linear mixed model summarized the relationship between the variation of male movement-based display and the season in male *P. vlangalii* lizards.

Tail coil	Variables	Estimate	SE	df	t value	*p*	Tail lash	Variables	Estimate	SE	df	t value	*p*
Duration	(Intercept)	1.047	2.838	54.680	0.369	0.714	Duration	(Intercept)	0.618	3.242	68.000	0.191	0.849
	Social context	0.066	0.125	40.414	0.532	0.597		Social context	-0.056	0.185	68.000	-0.302	0.764
	**Season**	**0.539**	**0.188**	**44.936**	**2.871**	**0.006**		Season	0.210	0.203	68.000	1.036	0.304
	Resident _SVL	0.096	0.062	54.677	1.542	0.129		Resident _SVL	0.059	0.071	68.000	0.826	0.412
	BT	0.004	0.014	43.747	0.288	0.775		BT	0.018	0.015	68.000	1.225	0.225
	Mass	-0.148	0.161	48.006	-0.922	0.361		Mass	-0.040	0.181	68.000	-0.218	0.828
CV of duration	(Intercept)	0.745	1.272	48.855	0.586	0.561	CV of duration	(Intercept)	-0.415	0.998	29.927	-0.416	0.681
	Social context	0.098	0.067	42.607	1.478	0.147		Social context	-0.020	0.057	27.062	-0.357	0.724
	Season	0.121	0.076	43.292	1.605	0.116		Season	-0.021	0.065	22.110	-0.323	0.750
	Resident _SVL	-0.004	0.027	48.695	-0.132	0.895		Resident _SVL	0.006	0.022	27.678	0.294	0.771
	BT	0.005	0.006	40.629	0.919	0.363		BT	0.006	0.005	19.176	1.329	0.199
	Mass	-0.030	0.065	44.330	-0.465	0.644		Mass	0.016	0.056	21.269	0.277	0.784
CV of mean amplitude	(Intercept)	-0.339	0.404	45.655	-0.837	0.407	CV of VT	(Intercept)	-0.021	1.677	32.380	-0.012	0.990
	Social context	-0.013	0.022	40.214	-0.613	0.543		Social context	-0.046	0.082	25.375	-0.558	0.582
	Season	0.039	0.024	40.314	1.636	0.110		Season	-0.039	0.112	26.887	-0.348	0.730
	Resident _SVL	0.004	0.009	45.444	0.460	0.648		Resident _SVL	-0.003	0.037	30.666	-0.094	0.925
	BT	0.003	0.002	37.458	1.854	0.072		BT	0.009	0.008	24.503	1.189	0.246
	Mass	0.020	0.021	41.081	0.987	0.329		Mass	0.066	0.097	26.127	0.678	0.504
CV of mean speed	(Intercept)	-0.451	0.620	43.886	-0.727	0.471	CV of mean speed	(Intercept)	-0.355	0.606	53.000	-0.586	0.560
	Social context	0.011	0.031	34.898	0.348	0.730		**Social context**	**-0.080**	**0.036**	**53.000**	**-2.199**	**0.032**
	Season	0.044	0.037	36.654	1.188	0.243		Season	-0.047	0.039	53.000	-1.206	0.233
	Resident _SVL	0.007	0.013	43.805	0.536	0.595		Resident _SVL	0.007	0.013	53.000	0.507	0.614
	**BT**	**0.007**	**0.003**	**34.026**	**2.637**	**0.013**		BT	0.003	0.003	53.000	1.079	0.286
	Mass	-0.008	0.032	38.388	-0.255	0.800		Mass	0.019	0.034	53.000	0.567	0.573
CV of maximum speed	(Intercept)	0.237	0.815	68.000	0.291	0.772	CV of maximum speed	(Intercept)	0.272	0.772	36.185	0.352	0.727
	Social context	0.042	0.045	68.000	0.944	0.348		Social context	-0.081	0.043	32.731	-1.890	0.068
	Season	-0.060	0.048	68.000	-1.253	0.215		Season	-0.032	0.051	29.251	-0.621	0.540
	Resident _SVL	0.008	0.017	68.000	0.481	0.632		Resident _SVL	0.000	0.017	34.240	0.009	0.993
	BT	-0.003	0.004	68.000	-0.836	0.406		BT	-0.001	0.004	26.299	-0.261	0.796
	Mass	-0.029	0.041	68.000	-0.698	0.488		Mass	0.018	0.044	28.389	0.404	0.689

CV, coefficient of variation; VT is defined as the volume of space swept by the tail tip during lashing in each display bout; Bold indicates statistically significant effects.

Table S2. The results of the general linear model summarized the relationship between the postpartum female's variation of movement-based display and parental care time in *P. vlangalii* lizards.

Tail dispiay	Variables	Estimate	SE	t value	*p*
Duration	(Intercept)	6.494	3.470	1.871	0.098
	**Parental care**	**0.063**	**0.023**	**2.746**	**0.025**
	Resident _SVL	-0.032	0.058	-0.543	0.602
	BT	0.015	0.043	0.360	0.728
CV of duration	(Intercept)	-1.503	1.506	-0.998	0.351
	Parental care	0.024	0.012	1.945	0.093
	Resident _SVL	0.025	0.027	0.909	0.393
	BT	0.018	0.021	0.863	0.417
CV of mean amplitude	(Intercept)	0.920	0.713	1.290	0.238
	**Parental care**	**0.014**	**0.006**	**2.402**	**0.047**
	Resident _SVL	-0.016	0.013	-1.242	0.254
	BT	0.001	0.010	0.124	0.905
CV of mean speed	(Intercept)	0.291	0.545	0.534	0.610
	Parental care	0.005	0.004	1.246	0.253
	Resident _SVL	-0.003	0.010	-0.343	0.741
	BT	0.001	0.007	0.193	0.852
CV of maximum speed	(Intercept)	1.253	0.626	2.001	0.086
	Parental care	0.005	0.005	1.025	0.340
	Resident _SVL	-0.017	0.011	-1.543	0.167
	BT	-0.002	0.009	-0.183	0.860

CV, coefficient of variation; Bold indicates statistically significant effects.

Table S3. The results of the general linear model summarized the relationship between the postpartum female's variation of movement-based display and reproductive investment in *P. vlangalii* lizards.

Tail dispiay	Variables	Estimate	SE	t value	*p*
Duration	(Intercept)	2.701	3.855	0.701	0.503
	**Relative litter weight**	**3.740**	**1.575**	**2.375**	**0.045**
	Resident _SVL	0.043	0.059	0.722	0.491
	BT	-0.006	0.044	-0.127	0.902
CV of duration	(Intercept)	-2.868	1.542	-1.860	0.105
	Relative litter weight	1.278	0.634	2.014	0.084
	**Resident _SVL**	**0.057**	**0.024**	**2.391**	**0.048**
	BT	0.002	0.018	0.114	0.913
CV of mean duration	(Intercept)	0.759	0.993	0.764	0.470
	Relative litter weight	-0.123	0.409	-0.301	0.772
	Resident _SVL	-0.002	0.015	-0.153	0.883
	BT	-0.010	0.012	-0.812	0.444
CV of mean speed	(Intercept)	0.014	0.584	0.024	0.981
	Relative litter weight	0.242	0.240	1.006	0.348
	Resident _SVL	0.004	0.009	0.427	0.682
	BT	-0.002	0.007	-0.334	0.748
CV of maximum speed	(Intercept)	0.958	0.649	1.476	0.184
	Relative litter weight	0.274	0.267	1.024	0.340
	Resident _SVL	-0.010	0.010	-1.013	0.345
	BT	-0.005	0.008	-0.655	0.534

CV, coefficient of variation; Bold indicates statistically significant effects.
